# Hypodermic Medication in Syphilis

**Published:** 1885-09

**Authors:** 


### Hypodermic Medication in Syphilis.

Prof. Streitz, in an article published in the *Rev. Biblograph, des Sciences Med.*, strongly advocates the treatment of syphilis by hypodermic medication. Hitherto, this treatment has been objectionable on account of the pain produced and the formation of abscesses. These abscesses are caused, first, when the injection is intracutaneous instead of subcutaneous; second, when the solution employed causes coagulation of albumen, and lastly, when foreign bodies are present in the solution. Two new formulas are given, which, he claims, obviate these difficulties:

| | | | |
|---|---|---|---|
| (1) ℞ | Hydrarg. Bichloride, - - | grs. v | |
| | Sodii Chloride, - - | dr. i.; grs. xv. | |
| | Aq. destillata, - - - | oz. v. | M. |
| | Sig. 10 to 20 minims, - | gr. 1-30 to gr. 1-15. | |

(2) *Peptonate of Mercury.—Bamberger:*

| | | |
|---|---|---|
| ℞ | Pepsini, - - - | grs. xv. |
| | Aq. distilla, - - - | oz. iij |
| | Hydrarg. Bichloride, - | grs. viij |

Re-dissolve precipitate by adding ammonium chloride grs. xl. After standing four days, this solution should be filtered. Dose, 5 to 10 minims.

